# Nanoscopy of bacterial cells immobilized by holographic optical tweezers

**DOI:** 10.1038/ncomms13711

**Published:** 2016-12-13

**Authors:** Robin Diekmann, Deanna L. Wolfson, Christoph Spahn, Mike Heilemann, Mark Schüttpelz, Thomas Huser

**Affiliations:** 1 Biomolecular Photonics, Department of Physics, University of Bielefeld, Universitätsstrasse 25, 33615 Bielefeld, Germany; 2 NSF Center for Biophotonics, University of California, 2700 Stockton Boulevard, Suite 1400, Davis, Sacramento, California 95817, USA; 3 Department of Physics and Technology, UiT The Arctic University of Norway, Klokkargårdsbakken 35, 9019 Tromsø, Norway; 4 Institute of Physical and Theoretical Chemistry, Johann Wolfgang Goethe-University Frankfurt, Max-von-Laue-Strasse 7, 60438 Frankfurt, Germany

## Abstract

Imaging non-adherent cells by super-resolution far-field fluorescence microscopy is currently not possible because of their rapid movement while in suspension. Holographic optical tweezers (HOTs) enable the ability to freely control the number and position of optical traps, thus facilitating the unrestricted manipulation of cells in a volume around the focal plane. Here we show that immobilizing non-adherent cells by optical tweezers is sufficient to achieve optical resolution well below the diffraction limit using localization microscopy. Individual cells can be oriented arbitrarily but preferably either horizontally or vertically relative to the microscope's image plane, enabling access to sample sections that are impossible to achieve with conventional sample preparation and immobilization. This opens up new opportunities to super-resolve the nanoscale organization of chromosomal DNA in individual bacterial cells.

## Abstract (web summary)

Nanoscopy of non-adherent cells is currently not possible, due to their movement in solution. Here the authors immobilize and manipulate fixed *E. coli* by multiple optical traps; their holographic optical tweezers enable *d*STORM imaging of orthogonal planes via 3D realignment of the sample.

To overcome the limit of diffraction in optical microscopy, several super-resolving microscopy or nanoscopy techniques have been developed in recent years^1^. A common caveat of all of these techniques is their extended data acquisition time. Localization microscopy techniques, such as direct stochastic optical reconstruction microscopy (*d*STORM) and photoactivated localization microscopy (PALM), typically require several thousands of image frames for the reconstruction of a single super-resolved image, leading to very long acquisition times on the scale of seconds to minutes^2^,^3^. This entails a high demand on the spatial position stability of the sample, which is typically achieved by chemical fixation and attachment of biological samples such as cells to glass coverslips.

Likewise, extended acquisition times make super-resolution microscopy inapplicable to non-adherent cells or other freely diffusing samples, such as bacterial cells or immune cells. Embedding these cells into gel matrices (for example, collagen or agarose) may alter their appearance and impair signal-to-noise ratios during the imaging process. In addition, the complex optical requirements for current nanoscopy methods restrict the ability to freely access and/or orient the sample with respect to the image plane or during cell–cell interactions, typically resulting in different spatial resolutions along different directions. Faster, parallelized versions of nanoscopy have been developed, but these still require the acquisition of several tens of images, making them too slow to image fast moving cells^4^,^5^,^6^,^7^. Indeed, non-adherent cells in suspension have not yet been imaged by any form of optical nanoscopy.

Here we show that it is possible to overcome these limitations in optical nanoscopy by the combined use of a holographic optical tweezers system and single-molecule localization microscopy. Beam shaping by a spatial light modulator (SLM) enables the generation and dynamic control of multiple, independent optical traps^8^. We combined this system with *d*STORM to facilitate the immobilization of the sample at specific, pre-defined positions and orientations in suspension during the nanoscopic imaging process.

## Results

### Influence of different optical trap powers on *d*STORM images

**Figure 1:**
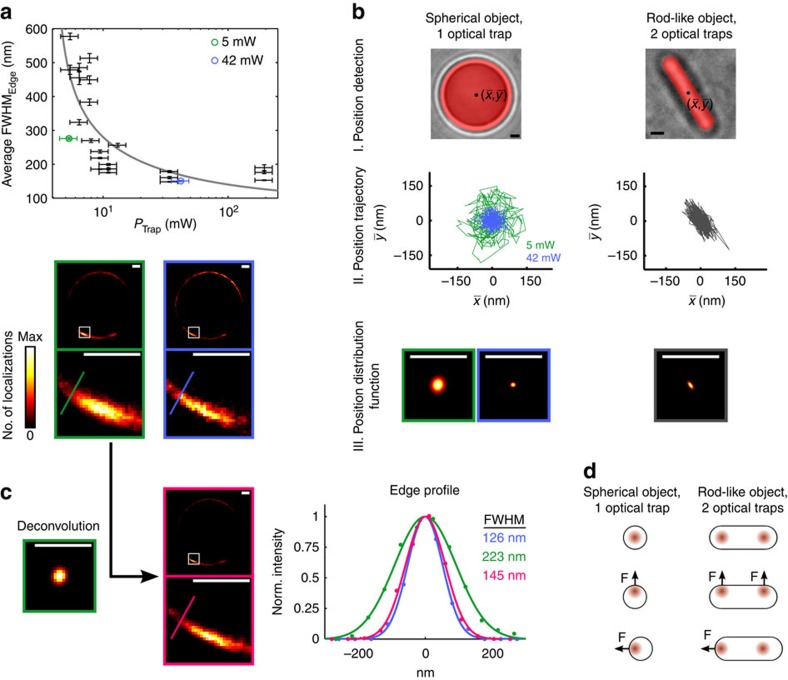
Response of the combined *d*STORM and optical tweezers setup. (**a**) Polystyrene beads (8.18 μm mean diameter) with fluorophores attached to their surface are optically trapped by applying different trapping laser powers. The average FWHM in the *d*STORM reconstruction of the bead edge is determined for the different trapping powers. For reference, the grey curve indicates the theoretically predicted power dependence. Lower images show *d*STORM reconstructions for trapping laser powers of 42 mW (blue) and 5 mW (green). Error bars indicate the 95% confidence interval for the FWHM and the variation of the optical trap power. (**b**) Principle of the PDF: (I) white light imaging of the trapped object (left: polystyrene bead, right: *E. coli* cell) followed by frame-by-frame determination of its ‘centre-of-mass'. (II) Typical position trajectories (5 s). Beads were trapped using 42 mW (blue) and 5 mW (green) laser power, revealing different extents of the trap stiffness according to **a**. (III) Multiple position determinations are used to calculate the PDF. In contrast to spherical beads, trapping rod-like-shaped bacteria results in a strongly elliptical PDF. (**c**) The PDF determined for a specific trap configuration is used for deconvolution, uncovering the much smaller structure of the bead's edge. The edge profiles for the same bead under modified conditions are normalized and a Gaussian function is fitted; for 5 mW of trapping power, FWHM values of 223 nm before PDF deconvolution (green) and 145 nm post-deconvolution (magenta) were determined, compared with a pre-deconvolution FWHM of 126 nm for 42 mW of trapping power (blue). (**d**) The ellipticity of the PDF is largely determined by the symmetry of the trapped object and the trap configuration. Displacements of equal amplitudes in either direction result in restoring forces on the bead of similar magnitudes towards the equilibrium position (left column). Trapping a rod-shaped object with two optical traps yields overall restoring forces of different magnitudes depending on the direction of the displacement (right column). Hence, the mean displacement amplitude is higher parallel to the long axis of the bacterium, which causes the PDF to be extended in that direction and results in the elliptical shape. Scale bars, 1 μm.

The potential of the combination of optical tweezers and *d*STORM (Supplementary Fig. 1) is demonstrated by first trapping microspheres in suspension. The surface of the microspheres is labelled with Alexa 647 for single-molecule localization by *d*STORM^9^. Owing to the short exposure time (29.55 ms), individual fluorophores on the bead surface, which are excited by highly inclined and laminated optical sheet illumination^10^, are in general localized with mean single-molecule localization precisions^11^,^12^ of ∼10–12 nm (Supplementary Fig. 2), mainly depending on the photon statistics of the fluorescence emission. This is comparable to what is typically achieved in localization microscopy of samples attached to a glass coverslip^13^. Nevertheless, holding the sample above the coverslip by optical traps weakens the effective localization precision due to position fluctuations of the sample (Supplementary Fig. 3). Depending on the laser power of the optical tweezers, the surface of the microspheres is reconstructed with different apparent widths in the *d*STORM images obtained from several thousands of image frames (Fig. 1a). Different near-infrared laser powers lead to differences in trap stiffness and, hence, to different restoring forces during the experiment. This circumstance becomes clear by considering an inherent property of optical tweezers: owing to weak interaction forces, an optically trapped object moves stochastically around a mean position, while experiencing Brownian motion of the surrounding molecules. As the optical trapping potential may be approximated by a harmonic oscillator potential^14^, the position of a trapped particle follows the statistics of a Gaussian distribution. Any fluorescent molecule that is firmly attached to the optically trapped particle will therefore exhibit the same displacement statistics. If the same single molecule is localized multiple times during the *d*STORM experiment, its localizations must exhibit the position distribution characteristics of the entire optically trapped sample (assuming the sample is stiff in comparison with the surrounding medium). The same applies to labelled structures that are visualized with subdiffraction resolution by single emitter localizations. Mathematically, this corresponds to the *d*STORM image of the structure, as it might be determined for an immobilized sample, convolved with the position distribution experienced within the optical trap. In case of the microsphere bead edges, this fact is illustrated by their average full width at half maximum (FWHM) becoming broader in the *d*STORM images as the trap power decreases and roughly following the theoretically expected dependence of 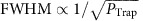 (Fig. 1a and Supplementary Note 1).

### Computational compensation of motion within optical trap

This consideration clearly bears analogies with the process of optical image formation where the diffraction-limited imaging process causes a structure to appear convolved by an optical point spread function (PSF) in conventional light microscopy. An established postprocessing technique in this case is image deconvolution^15^, exploiting the fact that by prior knowledge of the PSF (or even theoretical modelling of the PSF based on known parameters of the imaging system) the actual structure can be revealed from the originally recorded image more clearly. With regard to localization microscopy of samples held by optical tweezers, knowing the position distribution function (PDF) of the object inside the optical trap also enables the use of algorithmic deconvolution of the reconstructed image to reduce the influence of position fluctuations on the reconstructed image. This approach will hold if at least one of two conditions is met: (i) each individual label contributes several localizations to the reconstructed image or (ii) the distance between the labels is sufficiently small, that is, the imaged structure is densely labelled, which applies to the samples presented here (Supplementary Note 2).

**Figure 2:**
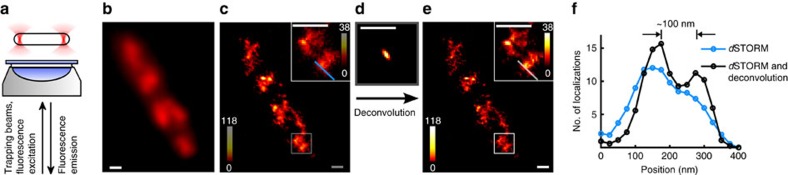
Super-resolution fluorescence microscopy of the chromosome of a fixed *E. coli* cell immobilized by two optical traps. (**a**) Schematics explaining the cell trapping by holding the bacterium using two optical tweezers at its end caps and the simultaneous super-resolution imaging experiment. (**b**) Diffraction-limited fluorescence image and (**c**,**e**) super-resolution fluorescence images before (**c**) and after (**e**) deconvolution using the PDF (**d** and Fig. 1b). Acquiring the raw *d*STORM data took ∼90 s. (**f**) The cross-sections shown in the insets demonstrate an increased spatial resolution of better than 100 nm for the labelled nucleoid structures following successful deconvolution. Scale bars, 500 nm.

Determination of the PDF is possible by detecting the trapped object's position frame by frame in a transmitted light image stack and binning its position trajectory to a two-dimensional (2D) grid (Fig. 1b). This can be done before or subsequent to the *d*STORM imaging, but in any case the trap configuration must not be altered between the imaging experiment and the determination of the PDF, that is, the number, relative arrangement and individual power of the optical traps need to remain unchanged (Supplementary Note 3). Using, for example, a centre of mass algorithm^16^, the PDF can also be identified for non-symmetrically shaped samples, such as single bacteria and other cells. Here, fixed rod-shaped *Escherichia coli* bacteria floating in suspension are captured by two optical traps, which are positioned at the cell's end caps (Fig. 2a). After the trajectory of the sample is binned, a 2D Gaussian function is fitted to the distribution and rendered to the same pixel size as the *d*STORM image for which it will be used during deconvolution (Methods).

With regard to the trapped beads, it becomes evident that the width of the PDF strongly depends on the trapping laser power and, therefore, the stiffness of the optical trap. Nevertheless, the outcome of the deconvolution process affirms that also in the case of low trap stiffness a similar result concerning an imaged structure is achieved in comparison with the same bead being trapped at 8.4 times higher power (Fig. 1c). This is of particular relevance for potential extensions to live-cell imaging, where preferably the lowest possible trap powers should be used to minimize phototoxicity^17^.

Comparison of trapped beads and bacteria shows that the shape of the sample and the trap arrangement also influence the PDF. The spherical symmetry of a bead held by one optical trap results in restoring forces largely independent of the direction of displacement in the focal plane (Fig. 1d) and hence an almost rotationally-symmetric PDF (Fig. 1b, left column). In contrast, trapping rod-like bacteria by two optical traps shows a dependence of the restoring force magnitudes on the direction of the displacement (Fig. 1b, right column). Both traps exert similar forces for a displacement orthogonal to the long axis of the bacterium (Fig. 1d). In case of a displacement parallel to the long axis, one trap becomes located in a region of the sample with a lower gradient in optical density. This leads to a decreased restoring force and weakens the overall trap stiffness, which results in a larger extent of the PDF in this direction and thus its ellipticity (Supplementary Note 3).

We note that as an alternative to the deconvolution approach, it might be possible to simultaneously track the position of the sample and acquire the *d*STORM data frame by frame. In this case, the position fluctuations could be used to directly correct for their effect on the localization data in each frame without the need for a statistical method. This could, for example, be realized by optically splitting the imaging path into two different colour channels, that is, one which images the sample position in a transmitted light colour channel and one which collects fluorescence signals for *d*STORM analysis. As a drawback, this approach would further complicate the optical setup, because the position detection by transmitted light and the fluorescence imaging path would now have to be split into two different channels, for example, separated by wavelengths. Furthermore, illuminating the sample with an additional wavelength could interfere with the photoswitching process. Thus, here we have used the less complex method of recording the statistical PDF and the *d*STORM data successively.

### Imaging optically trapped bacteria in solution by *d*STORM

Next, we applied this method to imaging chemically fixed (that is, dead) biological samples in suspension. *E. coli* bacterial cells are arranged parallel to the focal plane and held a few micrometres above the glass coverslip during data acquisition (Fig. 2a). Chromosomal DNA within the bacteria was pre-labelled with Alexa 647 using a click chemistry approach. Once trapped, a super-resolution image of the *E. coli* cell is recorded in ∼90 s. A comparison of the diffraction-limited fluorescence image (Fig. 2b) of the chromosomal DNA and the directly reconstructed *d*STORM image (Fig. 2c) readily demonstrates the power of super-resolution imaging of optically trapped biological samples. To verify that similar structures are observed in trapped and attached cells, *E. coli* cells are initially held in suspension and then pulled onto the coverglass by the optical traps. *d*STORM images recorded under these conditions show consistent structures in both cases (Supplementary Fig. 4). This observation indicates that in contrast to translational Brownian motion, no significant rotational Brownian motion appears in the case of the trapped bacteria. This is further verified by dividing the *d*STORM localizations of one trapped cell into separate reconstructions which, again, show consistent structures (Supplementary Fig. 5).

Deconvolution of the *d*STORM reconstruction (Fig. 2c) with the optical trap's PDF (Fig. 2d) leads to an image with increased spatial resolution and higher contrast (Fig. 2e). Structural features of the bacterial chromosome with dimensions of <100 nm can now be resolved (Fig. 2e,f). Close inspection of the super-resolution images reveals multiple nucleoids within a single cell. *E. coli* cells are governed by coupled replication and chromosome segregation, and exhibit overlapping replication cycles if grown in nutrient-rich media^18^. This results in multiple copies of the chromosome in each bacterial cell, which show different progress in their second replication cycle. In addition, it was recently shown that the nucleoids show heteromeric substructures, which are typical for a particular stage in the cell cycle^19^. Clearly, these heteromeric substructures of the *E. coli* chromosome are visualized in the super-resolution images of optically trapped *E. coli* (Fig. 2e).

### Multiple perspectives by controlling the sample orientation

**Figure 3:**
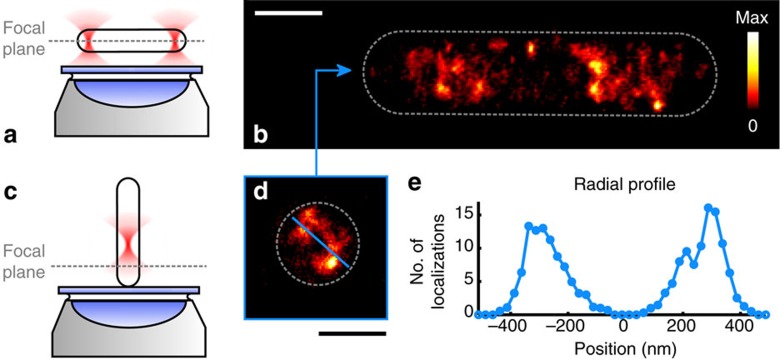
Holographic optical tweezers allow for super-resolution imaging of the same sample arranged in different orientations with respect to the focal plane. (**a**,**b**) Horizontal alignment reveals distinct structures of the labelled nucleoid DNA. (**c**,**d**) Vertical alignment indicates a heterogeneous distribution in the radial direction as well, with (**e**) decreased density near the centre of the cell. The approach of combining *d*STORM and optical trapping allows for isotropic super-resolution of 2D localization microscopy for each orientation of the sample, in this case with effective localization precisions of ∼28 nm for **b** and 31 nm for **d** (Supplementary Fig. 3). Recording the raw *d*STORM data took ∼90 s for **b** and 650 s for **d**. Scale bars, 1 μm.

The benefit of being able to adjust the sample's alignment relative to the image plane becomes apparent by our ability to consecutively image the same bacterium at different orientations: an *E. coli* cell is first held horizontally, that is, parallel to the focal plane (Fig. 3a), and *d*STORM data are recorded (Fig. 3b). Subsequently, one trap is switched off. This forces the bacterial cell to align vertically along the trap axis, caused by its rod-like shape following the form of the Gaussian laser beam, which propagates in the axial direction (Fig. 3c). The lower end cap of the *E. coli* cell is then pushed onto the coverslip, followed by a second acquisition of *d*STORM data (Fig. 3d). By this means, three-dimensionally (3D) super-resolved information of a specimen can be gathered, where each single image reaches the isotropic high resolution of 2D localization microscopy. This feature enables bypassing the inherently poorer spatial resolution of 3D localization microscopy along the optical axis. This is even more important in combination with fluorescent proteins, which emit substantially fewer photons than synthetic dyes and yield poor axial resolution in common 3D localization schemes. Still, for interpretation of the data, it must be taken into account that a reconstructed localization microscopy image shows a projection of the emitter's position in the axial direction within a certain volume around the focal plane that can span throughout the entire *E. coli* chromosome (Supplementary Fig. 6). Hence, aligning one axis of a sample parallel to the focal plane in a first step results in limited information about the radial emitter distribution around that axis (Fig. 3b), but this is achieved in the second step after aligning that axis orthogonal to the focal plane (Fig. 3d). The orthogonal alignment indicates that the nucleoid is located heterogeneously along the radial direction (Fig. 3d and Supplementary Fig. 7). Significantly lower DNA concentration is found near the centre of the bacterium, whereas DNA concentration (determined by the number of *d*STORM localizations) is increased at a distance of ∼200–380 nm from the centre.

## Discussion

The parallel alignment of *E. coli* reveals distinct heterostructures of the nucleoid along the long axis (Figs 2e and 3b, and Supplementary Fig. 7), which have been observed by *d*STORM before^19^. The reason for the tube-like structure of the nucleoid, which was revealed by measuring in orthogonal alignment, however, is still unclear (Fig. 3d and Supplementary Fig. 7). Nucleoids could be forced into this shape by radial confinement, but also the transertion model could explain the proximity to the membrane^20^,^21^. DNA compaction into thicker filaments is mediated by nucleoid-associated proteins^22^ and entropic effects. These filaments are believed to exhibit a bead-on-a-string formation with an estimated bead size of 130–440 nm (ref. 23). From our measurements, we determined a median size (FWHM) of the highly resolved DNA filaments of ∼85 nm in both parallel and orthogonal alignment of the cell (Supplementary Fig. 8), a value that is close to the lower bound of the reported bead size. To verify that the observed structures are neither artefacts caused by the labelling approach nor by handling the cells with the optical tweezers, identically prepared and additionally Sytox Green-labelled, but not optically trapped, *E. coli* cells were imaged by structured illumination microscopy (3D-SIM), allowing for 3D super-resolution imaging. Here, similar tube-like structures of the nucleoid are observed in planes parallel to the long axis of the cell (Supplementary Fig. 9). Although 3D-SIM provides the ability to observe multiple layers orthogonal to the focal plane and, hence, the long axis, it does not provide isotropic spatial resolution, because the axial resolution is approximately a factor of 3 lower than its lateral resolution^13^. A similar disadvantage also applies to common implementations of 3D localization microscopy, which also suffer from anisotropic resolution, usually worse in the axial direction^24^,^25^. With our combined HOT and *d*STORM system, this drawback is overcome by its ability to freely align samples with respect to the image plane. Although not providing the opportunity to render a full 3D image, realignment of the sample allows for uniform super-resolution imaging of conventional 2D localization microscopy in planes parallel and orthogonal to their respective axes. Immobilization and handling of suspension cells by optical tweezers is also readily compatible with other super-resolution imaging methods and opens up new opportunities to image and track cellular structures and interactions on the nanoscale.

## Methods

### Super-resolution microscopy

*d*STORM was performed on an inverted microscope (IX-71; Olympus) using an oil-immersion objective lens (PlanApo, × 60, numerical aperture (NA) 1.45 or Apo N, × 60 NA 1.49; Olympus; Supplementary Fig. 1). For fluorescence excitation, 488 and 647 nm laser light emitted by an argon–krypton ion laser (Innova 70C; Coherent) was selected by an acousto-optic tunable filter (AOTFnC-VIS-TN 1001; AA Opto Electronic) and additionally filtered by a bandpass filter (FF01–390/482/563/640-25; Semrock). Using two lenses (*f*=25 mm and *f*=120 mm), the beam was focused onto the back focal plane of the microscope objective lens. Behind the second lens, a mirror (BB02-E02; Thorlabs) was mounted on a linear translation stage to adjust the position of the beam entering the objective lens. *d*STORM experiments were performed in the highly inclined and laminated optical sheet mode, resulting in laser intensities of ∼5 kW cm^−2^ at the sample. The 647 nm laser line was used for the excitation of the fluorophore and conversion to the non-fluorescent triplet state of Alexa 647, whereas the 488 nm laser line was used for reversible photoswitching by recovery of the fluorescent singlet state. Fluorescence emission and white light images were magnified by a telescope and projected onto an EMCCD (electron-modifying charge coupled device) camera (iXon DV887DCS-BV; Andor) with a scale of 105 nm per pixel or 116 nm per pixel. For the selective suppression of excitation light and blocking of back-scattered light from the optical traps, three filters (LP02-647RU-25; Semrock, ET700/75 m; Chroma, FF01–775/SP-25; Semrock) were directly mounted to the camera. Alternatively, a white light source was used for position detection of the sample. For data acquisition, the software (Solis, Version 4.18; Andor) provided by the camera manufacturer was used.

3D-SIM was performed using a commercial OMX v4 Blaze system (GE Healthcare). The microscope was equipped with an oil-immersion objective lens (PlanApoN, × 60, NA 1.42; Olympus), a 488 nm excitation laser, a 528/48 emission filter and a scientific complementary metal-oxide-semiconductor (sCMOS) camera. Image stacks consisted of 15 images per 0.125 μm *z*-section, with full stack thicknesses of 1.75–3.0 μm.

### Holographic optical tweezers

For the generation of holographic optical tweezers, a 2 W diode-pumped all-solid-state infrared laser (MIL-H-1064; CNI) emitting light at a wavelength of 1,064 nm was used (Supplementary Fig. 1). The beam was expanded using a telescope (AC 254-030-B-ML, AC 254-100-B-ML; Thorlabs) before it was coupled through an objective lens (DIN 10, 10X, NA 0.25; Edmund) into a polarization-maintaining, high-power optical fibre (PMJ-A3HPM, 3S-1060-6/125-3AS-3; OZ Optics). The global infrared laser power was controlled with an adjustable neutral density (ND) filter wheel. Using either a lens (AC 508-200-B-ML; Thorlabs) in combination with an adjustable aperture for spatial filtering (not shown in the sketch) or a collimator (F810SMA-1064; Thorlabs), the infrared beam was expanded to overfill the active area of a SLM (XY Series 512 × 512; Boulder Nonlinear Systems). A 4*f*-telescope consisting of two lenses (50 mm diameter, AC508-300-B-ML; Thorlabs) imaged the SLM onto the back focal plane of the microscope objective lens (PlanApo, × 60, NA 1.45; Olympus). The laser tweezers' light path was overlaid with the fluorescence excitation light path using a dichroic mirror (NFD01-1064-25x36, Semrock). To spectrally filter the excitation and trapping light from the fluorescence emission, a second dichroic mirror (FF502/670-Di01-25x36x3.0, Semrock) was used. A folding mirror inside the microscope body (not shown in the sketch) allows the imaging path to be guided through a short-pass filter (E700SP-2P; Chroma) to a CMOS camera (UI-1240SE-NIR-GL CMOS; IDS) (not shown in the sketch), which was used solely for alignment and video feedback for the software controlling the optical trap pattern, but not for super-resolution image acquisition. The camera and the SLM were connected to a computer (Intel i5–2400 3.1 GHz CPU, 4 GB RAM, Windows 7, 32-bit OS) equipped with a GPU (GeForce GTX 550Ti; Nvidia) for real-time computation of the phase pattern applied to the SLM. LabVIEW (LabVIEW 2010 SP1; National Instruments) software^26^,^27^ was used to control the trapping experiment and to compute the phase pattern corresponding to the traps' positions either by a lens-and-prism-phase or Gerchberg–Saxton-based algorithm.

### Estimation of optical trap power

The power of the individual optical traps was controlled in two ways: The global power provided by the SLM was determined by adjusting the neutral density filter wheel and measuring the power reflected from the active SLM area. In addition, the 0th diffraction order (that is, the unmodulated part of the beam) was used as a power sink to effectively decrease the power of the holographic traps that were always created in locations different from the 0th diffraction order. To estimate the individual trap powers, we conducted a reference measurement by placing a small aperture in the focal plane behind the first lens of the 4*f*-telescope, which is conjugate to the sample plane. This aperture was adjusted to block all infrared laser light from the SLM, except for one optical trap. The corresponding optical power of this single optical trap was measured by placing the sensor of a power meter at the position of the objective lens. Measurements were conducted for 45 different values of the relative sink power of the 0th diffraction order in combination with an overall number of one, two, three or four holographically created optical traps, as no more than four optical traps were simultaneously used throughout this work. From this table of 45 measured trap power values for each overall number of either one, two, three or four of the holographic optical traps, a cubic spline interpolation was used to estimate the effective single optical trap power for the sink power settings used in the according experiments.

### Image acquisition

For the reconstruction of the super-resolved fluorescence images of microsphere samples obtained by *d*STORM, ∼1,500 to 12,000 frames were acquired at an exposure time of 29.55 ms per frame, with a detector temperature of −70 °C and an EMCCD gain setting of 200. As many measurements of the same sample were conducted in short succession, the number of frames necessary to achieve a sufficient number of localizations increased for later acquired data due to photobleaching. Hence, the acquisition time for these images ranges from ∼50–400 s. In the *E. coli* measurements, 8,000–66,000 frames were acquired with an exposure time of 4.64–8.24 ms, a detector temperature of −70 °C and an EMCCD gain setting of 200. Again, successive measurements of the same sample result in an increased number of frames for the second acquisition. The according acquisition times range from ∼90 s for the parallel alignment relative to the focal plane and up to 650 s for the orthogonal alignment.

White light image stacks were recorded before or after *d*STORM data acquisition. These consist of ∼1,500–10,000 frames with a camera exposure time of 4.64–29.55 ms per frame. To achieve a higher contrast for the automated position detection used to determine the PDF, the bacteria were slightly displaced (∼700 nm) along the axial direction relative to the imaging plane by adjusting the axial position of the optical traps. The positions of the traps relative to each other were not changed and no significant changes in the movement of the bacteria were observed.

### *d*STORM image processing and analysis

The open-source software *rapid*STORM^28^,^29^ (Version 3.2 and Version 3.3) was used for single emitter fitting of the raw *d*STORM data. The output files containing the detected emitters' coordinates were used as inputs for a custom-written MATLAB programme to generate image files with adjustable pixel size and pixel values directly proportional to the number of detected single emitters by using linear interpolation. All *d*STORM reconstructions of *E. coli* cells used a pixel size of 25 nm × 25 nm, whereas all *d*STORM reconstructions of microspheres used a pixel size of 50 nm × 50 nm. Further analysis and processing was done either directly in MATLAB or in the open-source software Fiji^30^. The colour tables for displaying the images were chosen such that appropriate contrast was achieved and all colour bars were linearly scaled in terms of localization density, that is, localizations per pixel. In the case of *E. coli* bacteria attached to the coverslip (Supplementary Fig. 4c), custom-written MATLAB code was used for automated drift correction^31^.

### Algorithmic data evaluation

To automatically measure the average FWHM of the edges of the trapped beads (Fig. 1a), custom-written MATLAB code was used: The centre of the bead was first determined algorithmically, followed by a transformation to polar coordinates around the centre and an averaging step over all angles. The FWHM of the edge was computed by fitting a Gaussian function to the average radial profile of the *d*STORM emitter localizations. A similar procedure without the fitting step was carried out to determine the radial intensity distributions for the labelled *E. coli* nucleoids (Supplementary Fig. 7).

### PDF generation and image deconvolution

To obtain the PDF of a trapped object, the position was detected in several transmitted light image frames using a custom-written MATLAB script based on a centre of mass algorithm^16^: Thresholding was applied to slightly smoothed images to create a filtering mask, which was overlaid with the raw image data. The centre of mass of the region inside the mask was computed according either to the pixel values in a normalized, background-subtracted image or a binary image to obtain the object's position. A 2D histogram was generated from all detected positions in one image stack. An elliptical, 2D Gaussian function (Supplementary Note 1) was fitted to this deviation. The function was normalized and stored in an image file as PDF. The PDF pixel size was chosen to match the pixel size of the *d*STORM image for which it was used during the subsequent deconvolution process, which was performed using the Richardson–Lucy algorithm implementation of the ImageJ/Fiji plug-in DeconvolutionLab (see Vonesch, C., Terrés Cristofani, R. and Schmit, G. DeconvolutionLab http://bigwww.epfl.ch/algorithms/deconvolution).

### Microsphere labelling

The streptavidin-coated surface of superparamagnetic microspheres (CM01N, Bangs Laboratories) with a mean diameter of 8.18 μm was labelled with Alexa 647 bound to a single-stranded, 10 nucleobases-long DNA strand functionalized with biotin (IBA). Four microlitres of bead stock solution and 8 μl of 10^−6^ M solution of the labels were added to 400 μl bi-distilled water containing 0.01% (v/v) Tween 20 and vortexed at ∼1,000 r.p.m. at room temperature for ∼30 min. Subsequently, the beads were pulled to the bottom of the reaction tube by a permanent magnet and washed three times with water containing 0.01% (v/v) Tween 20 to remove unwanted residuals. After the last washing step, the tube was filled with 170 μl of PBS with 0.15% (w/v) BSA (Sigma Aldrich). Forty microlitres were mixed with 55 μl imaging buffer (10 μl of 1 M mercaptoethylamine (Sigma-Aldrich) with 45 μl of oxygen scavenger system solution^9^). Sixty microlitres of the final solution was applied to a sealed chamber (mostly to avoid the external induction of currents, for example, by air movement) and used for the experiments.

### Labelling of chromosomal DNA in *E. coli*

The *E. coli* strain MG1655 was inoculated from glycerol stocks and grown overnight in lysogeny broth (LB) medium (Sigma) at 32 °C, while shaking at 200 r.p.m. Working cultures were inoculated 1:200 in lysogeny broth medium from overnight. 5-Ethynyl-2′-deoxyuridine (EdU) (10 μM; Baseclick) was added for 40 min at an OD_600_ of ∼0.25, to cover the time needed for one complete replication round. As up to three or four replication rounds occur concomitantly at fast growth rates (here: 27 min doubling time)^32^ and chromosome replication requires ∼40 min at doubling times shorter than 60 min (ref. 33), 40 min of exposure to EdU are sufficient to label the entire chromosome, even if the primary replication round has already replicated most of the chromosome. After fixation with 1% formaldehyde in phosphate buffer, cells were centrifuged at 5,000 *g* for 5 min and washed twice in 100 mM phosphate buffer (pH 7.4). The click reaction was performed as published previously^19^. Cells were washed thrice with PBS (Sigma), centrifuged and kept as stock solution. A volume of 20 μl of 20% (v/v) of the *E. coli* stock solution in PBS was mixed with 55 μl of imaging buffer and 60 μl of this solution was used in a sealed chamber for the optical trapping and *d*STORM experiments. For 3D-SIM imaging, the *E. coli* stock solution was additionally labelled with 3.3 μM Sytox Green (ThermoFisher) for 5 min. This cell solution was immediately mixed with an equal volume of mounting media consisting of Vectashield H-1000 (Vector Laboratories) with 8.6 mM each of methyl viologen (Aldrich) and ascorbic acid (Fluka), before being mounted onto a slide and sealed from the external environment.

### Data availability

All data and processing steps supporting the findings of this study are available within the article and its Supplementary Information files, or can be requested from the corresponding author upon request, including raw *d*STORM data and scripts for analysis.

## Additional information

**How to cite this article:** Diekmann, R. *et al*. Nanoscopy of bacterial cells immobilized by holographic optical tweezers. *Nat. Commun.* **7,** 13711 doi: 10.1038/ncomms13711 (2016).

**Publisher's note:** Springer Nature remains neutral with regard to jurisdictional claims in published maps and institutional affiliations.

## Supplementary Material

Supplementary Information Supplementary Figures 1-11, Notes 1-3 and References 1-18
