# Supplementary material for: Nanoscopy of bacterial cells immobilized by holographic optical tweezers
Source: Nat Commun. 2016 Dec 13;7:13711. doi: 10.1038/ncomms13711 (PMC5159804; doi:10.1038/ncomms13711)
Supplement: Supplementary Information — Supplementary Figures 1-11, Notes 1-3 and References 1-18 [file ncomms13711-s1.pdf]

Schematics of the combined optical tweezers and single molecule localization microscopy setup

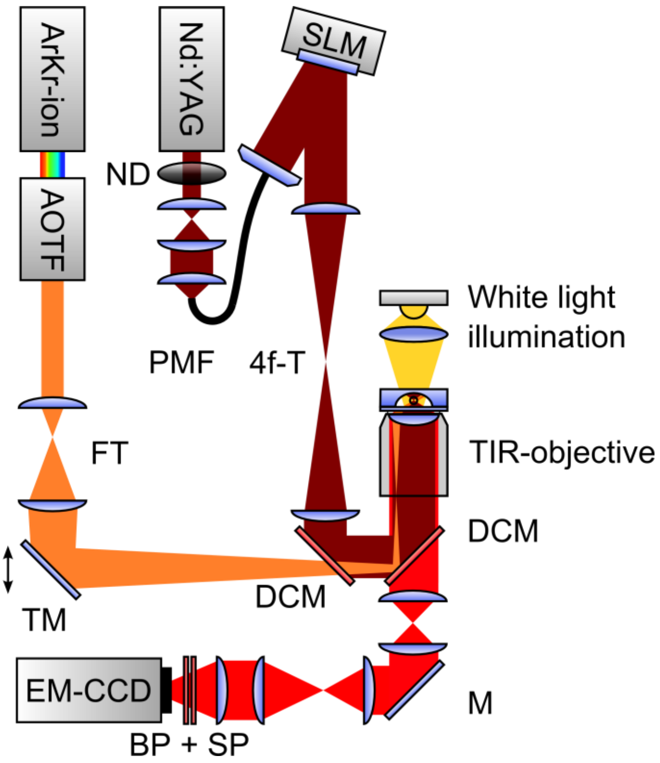

Supplementary Figure 1: Layout of the *d*STORM setup equipped with holographic optical tweezers for immobilizing free-floating cells: Excitation light is emitted from an Ar-Kr-ion laser and selected by an AOTF. For the optical tweezers implementation, a 1064 nm wavelength laser beam is emitted from a Nd:YAG laser and expanded to fill the optically-active area of a spatial light modulator (SLM), which displays a phase pattern according to the arrangement of the optical traps within the software. Both the excitation and the trapping light are coupled into the same objective. *d*STORM measurements are performed in HILO mode. For position detection of the sample, a white light source is used. The fluorescence emission and the white light illumination are spectrally filtered and imaged using an EM-CCD camera. Abbreviations: AOTF – acousto-optical tunable filter, FT – focusing telescope, TM – translatable mirror, DCM – dichroic mirror, NA – numerical aperture, TIR – total internal reflection, ND – neutral density filter wheel, PMF – polarization-maintaining fiber, 4f-T – 4f-telescope, M – mirror, BP – band-pass filter, SP – short-pass filter.

Single molecule localization precision of individual fluorophores attached to optically trapped microspheres

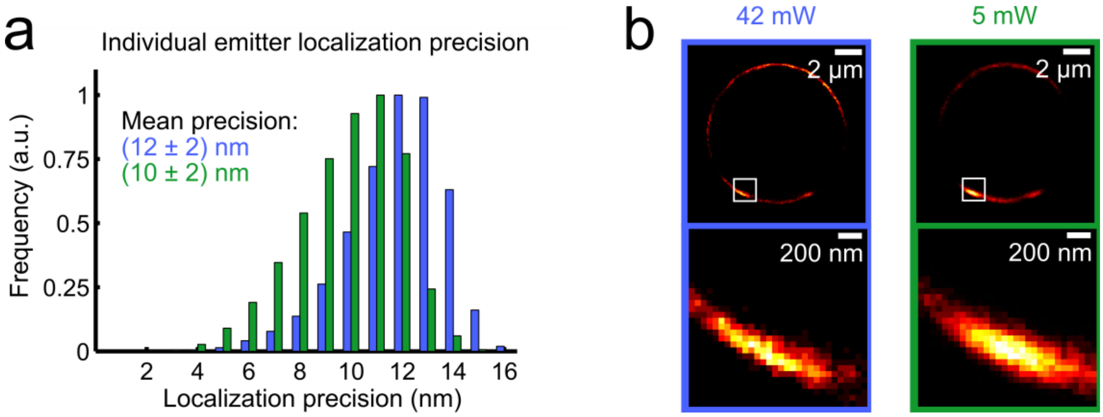

Supplementary Figure 2: (a) Histogram of the individual single molecule localization precisions[1] of Alexa 647 dyes attached to the surface of an 8.18 µm bead (b). The bead is held a few µm above the coverslip by optical traps with trap powers of about 42 mW (blue) and 5 mW (green), respectively. The mean single molecule localization precisions are (12 ± 2) nm (for 42 mW trapping laser power) and (10 ± 2) nm (for 5 mW trapping laser power). The data for 42 mW trapping laser power was recorded prior to the data for 5 mW trapping laser power. This explains the slightly better values for the single molecule localization precisions in the latter case which result from decreased background noise due to a lower number of remaining active fluorophores around the focal plane.

Values for the mean single molecule localization precisions were determined by fitting a Gaussian function to the data and are given using the distances to the 95 % confidence bounds of the according fit parameter as the error interval.

Effective localization precision along different axes of bacterial cells

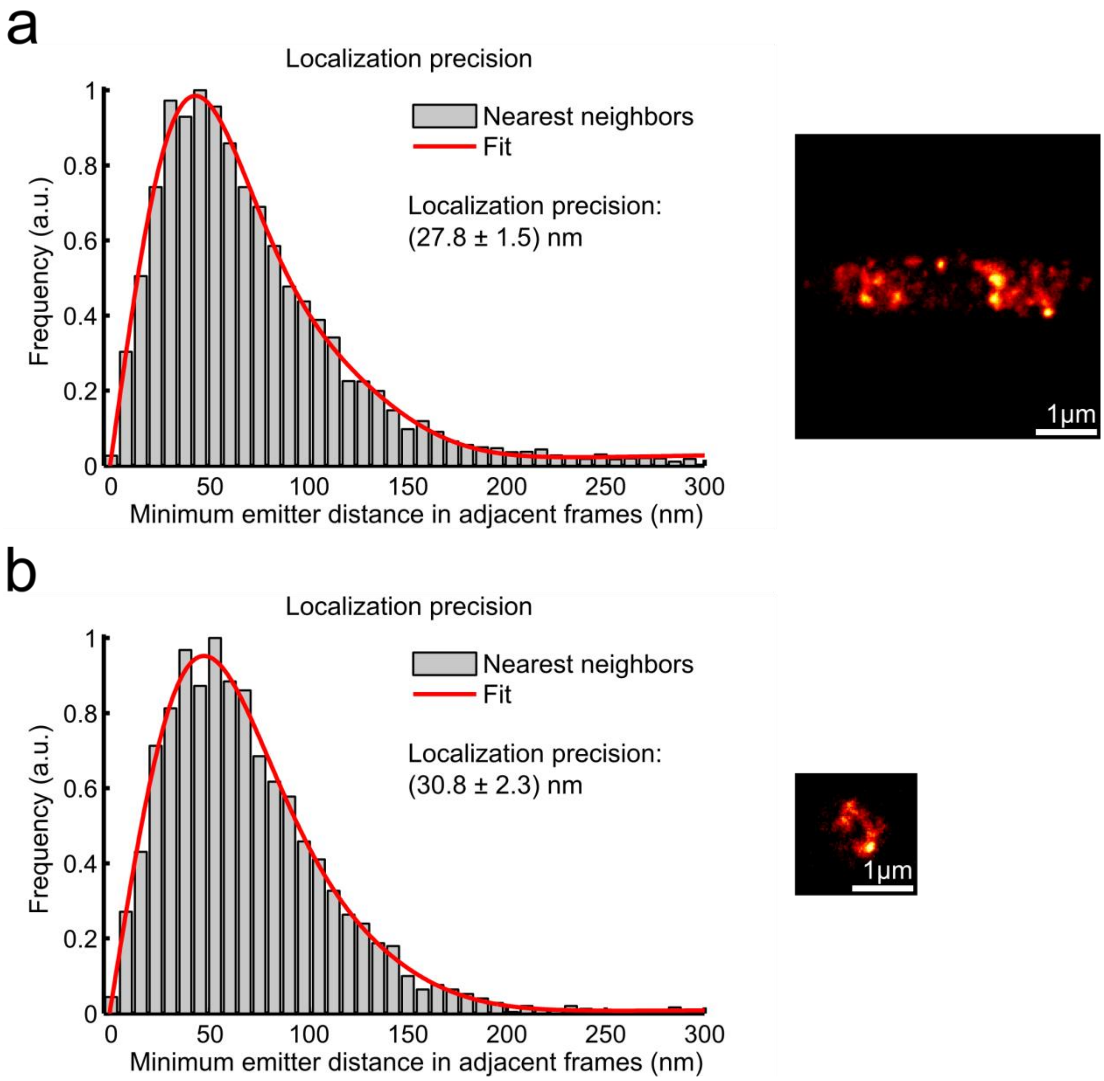

The average effective localization precision of the *d*STORM experiments (**Fig. 3**) is estimated using the method proposed by Endesfelder et al.[2]. This approach determines the localization precision from the minimum distance between localized emitters in adjacent frames. It is therefore capable of considering both the influence of the finite single molecule emitter localization precision[1,3] as well as the position fluctuations inside the optical trap. Accordingly, these values represent the effective, average localization precision of *d*STORM images of optically trapped samples. The corresponding values are (27.8 ± 1.5) nm for the alignment parallel to the focal plane (a) and (30.8 ± 2.3) nm for the alignment orthogonal to the focal plane (b). Values for the effective localization precisions were determined by fitting the model of Endesfelder et al. to the data and are given using the distances to the 95 % confidence bounds of the according fit parameter as the error interval.

Comparison of *d*STORM images of bacterial cells while trapped in suspension by optical tweezers and after pull-down onto a surface.

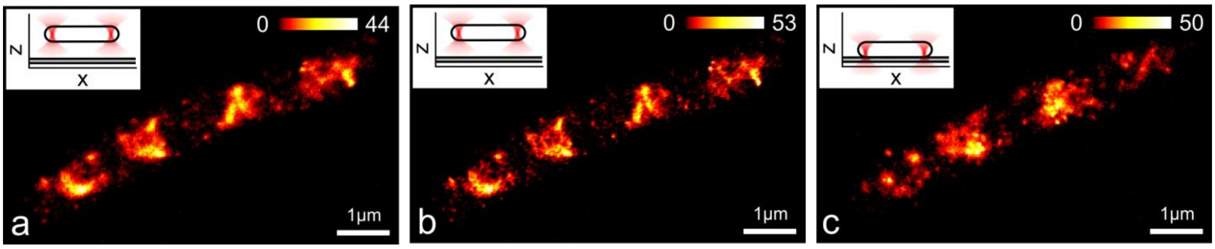

Supplementary Figure 4: Super-resolution images of the same *E. coli* during optical trapping and after deposition on a substrate: (a) *d*STORM image of the optically trapped bacterial cell, (b) deconvolved *d*STORM image of the optically trapped cell, and (c) *d*STORM image of the cell attached to a coverslip. Direct comparison shows that similar structures can be observed in the optically trapped *E. coli* cell in solution compared to the same cell stuck to the cover slip. Subtle differences are likely due to rotation of the cell during deposition onto the substrate and bleaching as the images were acquired subsequently.

Rotational Brownian motion

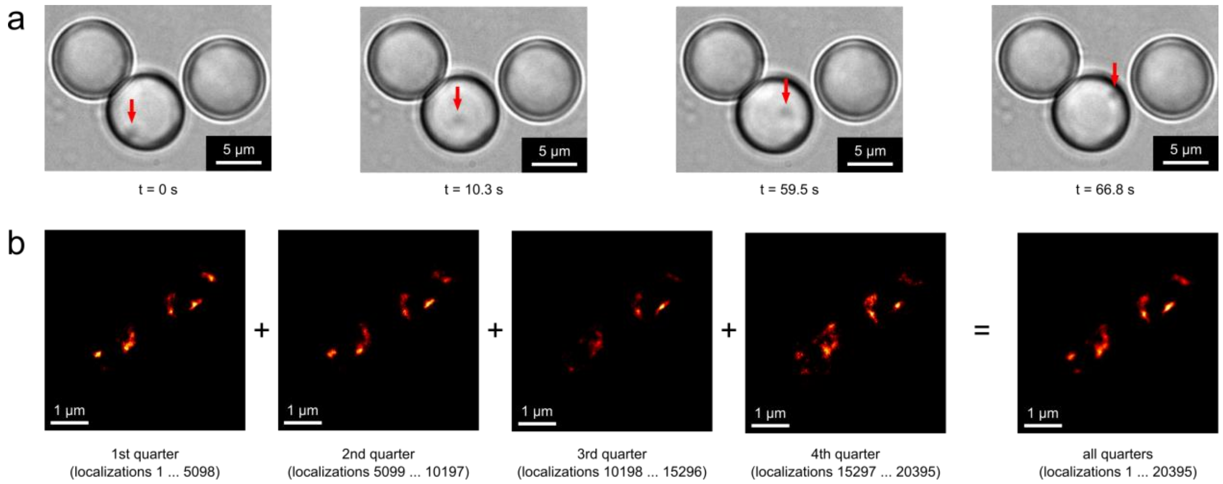

Supplementary Figure 5: Rotational Brownian motion inside the optical traps is evaluated using a series of white light images (a) and *d*STORM images (b). Image sequence (a) shows three 8.18 µm sized polystyrene beads of which each is held by one optical trap with individual trap powers of (9.9 ± 1.6) mW. The two beads on the left are stuck together, whereas the bead on the right can move independently. The red arrow marks a structure on a bead in the middle. This structure changes its position over time and shows a rotation of the two beads inside the traps around their common axis. This is possible as the beads possess an almost homogeneous inner structure and present a rotation symmetric formation, such that the optical traps do not restrict rotational motion. In contrast to the trapped beads, no obvious structure is visible in the whitelight images of the trapped *E. coli* bacteria. Hence, we use a series of *d*STORM images to evaluate the rotational motion of a bacterial cell held by two optical tweezers at its end caps (b). 20,395 localizations in 15,224 raw images are split up into 4 quarters with an equal amount of localizations in each (four images on the left). Their sum results in the complete *d*STORM reconstruction (right image). Comparing the quarters with the complete reconstruction shows that similar structures are observed in each image while subtle differences presumably result from the stochastic nature of the *d*STORM data acquisition. Hence, we conclude that in contrast to the beads, the *E. coli* cells do not exhibit rotational Brownian motion inside the optical traps during the *d*STORM imaging process which in this case took about 90 s. We assume that rotational motion is frustrated by the interaction of the elliptically shaped trap focus with the inner structure of the cells that features small refractive index gradients throughout parts of the cellular volume. From these observations we conclude that rotational Brownian motion does not significantly affect the *d*STORM images of the optically trapped bacteria.

Estimation of the imaged z-range

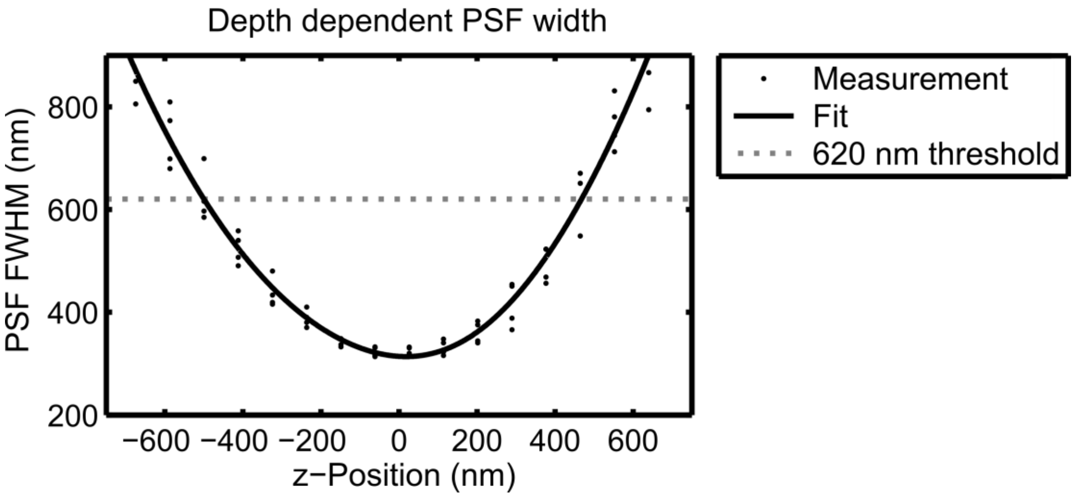

Supplementary Figure 6: As the 2D *d*STORM images show a projection of the emitter distribution in a volume around the focal plane, we use information from the detected PSF widths to estimate the range in z-direction that contributes to the reconstructed image. For a reference measurement, fluorescent Tetraspeck beads with a diameter well below the diffraction limit (100 nm diameter (Invitrogen)) are immobilized in double distilled water to the bottom of a Nunc Lab-Tek II Chamber Slide System (ThermoFisher). Using a z-piezo stage, the sample is moved in steps of 100 nm through the focal plane. We use *rapid*STORM to detect the FWHM of the PSF in one direction (measurement points in the plot) and fit the depth dependent PSF model of Huang et al.[4] (solid black curve). The z-position was corrected for the focal shift[5] induced by the refractive index mismatch of the water/glass interface (n_{water}/n_{glass} = 1.33/1.518). The raw data of the *d*STORM image shown in **Fig. 2c** was again analyzed with *rapid*STORM using identical settings except for one modification: a fixed setting for the PSF width was used for the reconstruction shown in **Fig. 2c**, while we allow *rapid*STORM to consider the PSF width as a free fitting parameter for the depth analysis. Consequently, more localizations are found because less restrictions are imposed on the fitting routine (38,828 instead of 22,438 localizations). To filter physically reasonable values, all localizations with a PSF FWHM below 200 nm are rejected. The upper bound is chosen such that the same number of 22,438 localizations as contributing to the reconstruction of **Fig. 2c** is kept. The according upper bound for the PSF FWHM is 620 nm. We conclude that emitters with PSF FWHM values of up to approximately 620 nm (dotted gray line) contribute to the reconstruction of the *E. coli* cell shown in **Fig. 2c.** The plot shows that this corresponds to emitter z-positions ranging from about -500 nm to 470 nm, i.e. covering an interval of approximately 970 nm. The diameter of the *E. coli* cells is on the order of 950 nm as measured from the transmitted light images. It follows that the entire *E. coli* chromosome can contribute to the 2D projection constituting the *d*STORM reconstruction if the focal plane is placed in the center of the bacterial cell.

Distribution of chromosomal DNA in several different *E. coli* cells

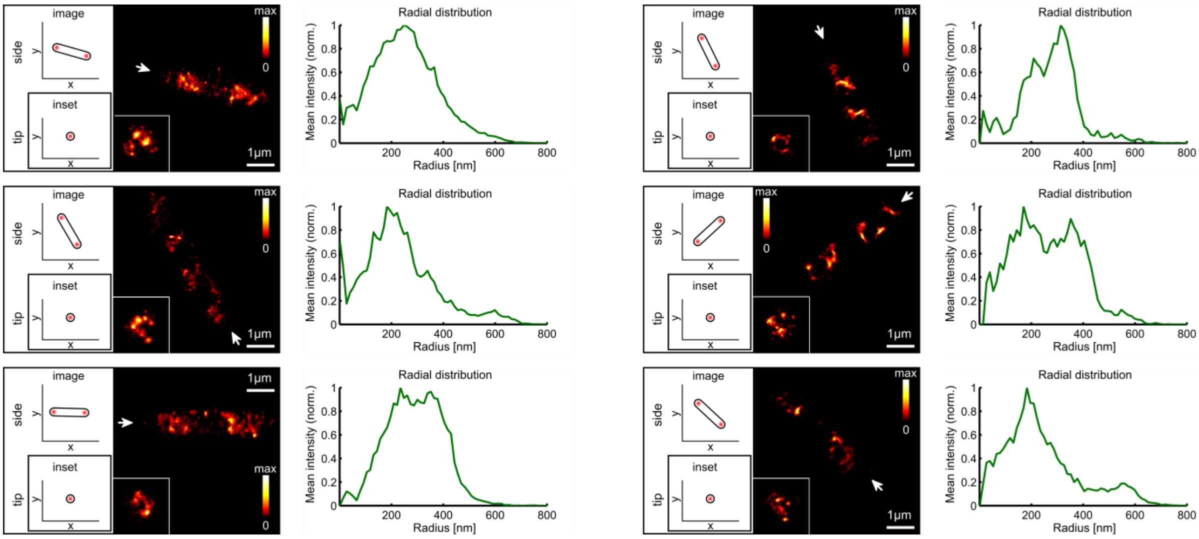

Supplementary Figure 7: *d*STORM images of Alexa 647-labeled nucleoids in optically trapped *E. coli* cells, imaged using parallel alignment (large images) and orthogonal alignment (insets) to the imaging plane. For orthogonal alignment, the marked tips are pulled onto the coverslip. The different numbers of distinct nucleoid structures represent different stages within the cell cycle, as also indicated by the cell length[6]. In either case, the density of the chromosomal DNA (determined from single molecule localizations) decreases towards the center of the bacterium (plots) in radial direction. Curves show the radial density distribution, averaged over all angles. Note that a radius of 0 nm corresponds to the center of the bacterium and the averaging step was chosen in order to obtain a more objective measure of the radial distribution in comparison to selecting one specific radial profile.

Observed widths of the chromosomal DNA structures in *E. coli* cells

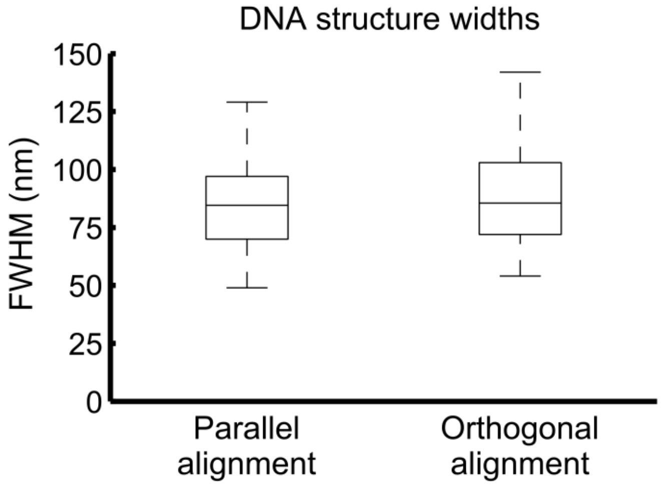

Supplementary Figure 8: Box plot of the structure widths of the chromosomal DNA for the bacterial cells shown in **Supplementary Fig. 7**. Following the approach of *Wegel et al*[7], we measured the apparent DNA structure widths. Using Fiji[8], five line-profiles of 50 nm line width were measured for each cell at different locations on the chromosomal DNA filaments, both for the parallel and orthogonal alignment to the focal plane. The full width at half maximum (FWHM) was determined by fitting a Gaussian function to each line profile. The median chromosomal DNA filament width measured is 84.5 nm for the parallel alignments (n = 30) and 85.5 nm for the orthogonal alignments (n = 30) relative to the image plane.

Distribution of chromosomal DNA in *E. coli* cells as measured by super-resolution 3D structured illumination microscopy for a complementary labeling approach

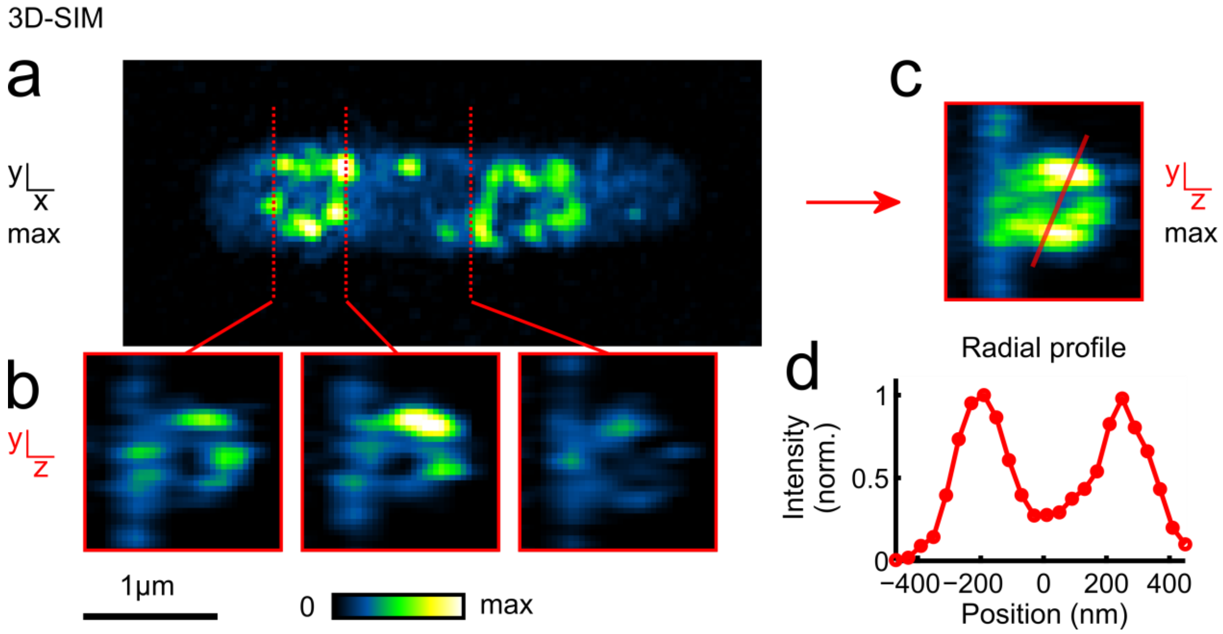

Supplementary Figure 9: Three-dimensional structured illumination microscopy (3D-SIM) shows that similar structures of chromosomal DNA are observed from *E. coli* cells on substrate as compared to optically trapped cells (**Fig. 3**). This holds both for the lateral (a) and axial (b,c) distribution of the observed chromosomal DNA. Also, the radial intensity profile (d) reveals a similar radial distribution as found using the *d*STORM approach (**Fig. 3e**).

3D-SIM images show the fluorescent signal of Sytox Green, which was used to post-label identically prepared bacterial cells (**Methods**). These images indicate that the observed tube-like structures do not originate from the click-chemistry labeling approach as they are also observed using this alternative strategy. (a) shows a maximum intensity projection (excluding the slices with strong signal from the coverslip to avoid masking of the chromosomal structure) of the bacterial cell with the long axis lying in the focal plane, while (c) shows a maximum intensity projection with the long axis orthogonal to the image plane. (b) shows single slices orthogonal to the long axis.

Displacement of an optically trapped polymer bead by highly inclined laminated sheet illumination

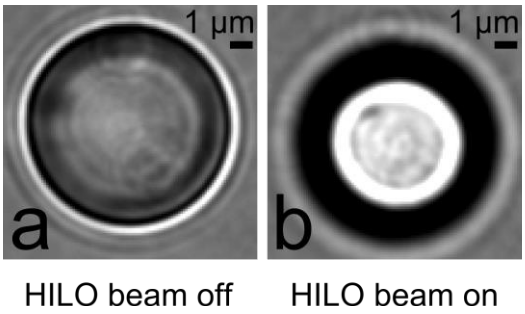

Supplementary Figure 10: Displacement of an optically trapped object by additional oblique laser illumination: (a) A bead is trapped with a mean lateral trap stiffness of κ = (1.5 ± 0.2) pN/µm, as determined by the power spectrum of its trajectory in 2,162 white light frames[9], while the HILO-beam for fluorescence excitation and photoswitching is turned off. By switching the HILO beam on (b), the lateral equilibrium position is shifted by (750 ± 28) nm. Furthermore, an axial shift occurs, clearly indicated by the defocused view. This fact emphasizes the need for carefully setting all parameters influencing the object position inside the optical traps (and, if necessary, adjusting these parameters, e.g. in the case of defocusing due to the HILO beam) before the measurement data is recorded. The trap stiffness was determined from fitting the according model to the power spectrum, using the distances to the 95 % confidence bounds of the according fit parameter as the error interval. The shift of the lateral equilibrium position is given as the shift of the center-of-mass determined from the white light images and the error is calculated by Gaussian error propagation.

Fit of the position distribution function (PDF)

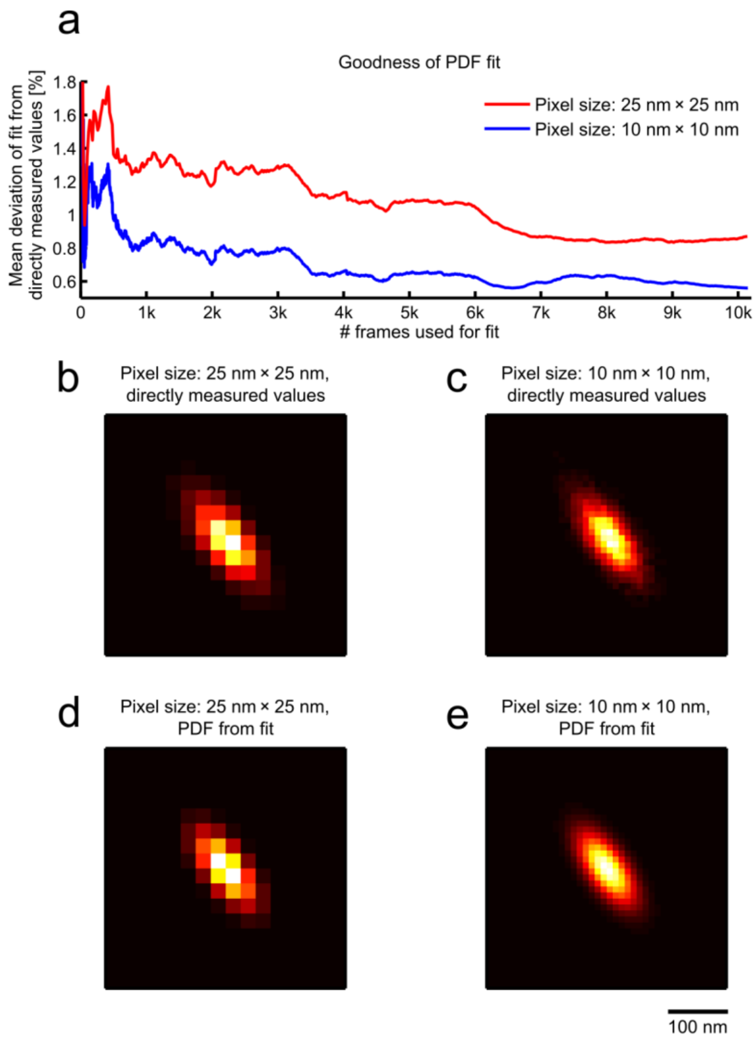

Supplementary Figure 11: A white light image stack of the sample shown in **Fig 2, Supplementary Fig. 4**, consisting of 10,146 frames, is used to evaluate how many measured frames are necessary to generate a PDF with sufficient accuracy. Therefore, only a certain number of frames starting from the first frame are used for fitting the 2D elliptical Gaussian, and the result from the fit is compared to the 2D histogram, which is directly obtained from the position measurements of all 10,146 frames. Using a pixel size (according to a bin size) of 25 nm × 25 nm, the mean deviation from the fit (d) to the measured values (b) of all 10,146 frames drops below 1.4 % (within the central region of 400 nm × 400 nm) if data from more than 500 frames is used for the fit (a). The PDF accuracy is even further increased by using a smaller pixel size of 10 nm × 10 nm (c,e). This leads to the presumption that the deviation results in large part from undersampling: in the case of the measured data, the value of a bin effectively reflects the mean of the deviation of all points inside the bin, while in case of the fit, the value of the bin is computed only from the point at its center, neglecting the shape of the distribution inside the

residual bin. Using relatively large pixels, the accuracy might be increased by introducing an oversampling step, i.e. computing the function values on a finer grid, and averaging them afterwards. Most importantly, it has to be considered that the resulting PDF exhibits the same pixel size as the *d*STORM image for which it is used during the deconvolution. The excellent agreement between the directly measured values (b,c) and the PDF obtained from the fit to all available frames (d,e) shows that an elliptical 2D Gaussian is a valid model to describe the position distribution of optically trapped objects. This is also valid for non-spherical samples and multiple trap foci as both are present in case of the *E. coli* held by two traps.

Supplementary Note 1: Position fluctuations inside an optical trap.

The position distribution of an optically trapped particle can be approximated by the function

$$\mathrm{PDF}(\mathbf{x}) = A \exp\left\{\frac{-\mathrm{V}(\mathbf{x})}{k_B T}\right\},$$

where $k_\mathrm{B}T$ is the thermal energy, $\mathrm{V}(\mathbf{x})$ is the trapping potential, and A is a normalization factor[10,11]. The trapping potential can be approximated by a harmonic potential in the vicinity of a stable equilibrium position, which is usually assumed for optical traps[12]. Hence, the restoring force is

$$\mathbf{F}(\mathbf{x}) = -\kappa(\mathbf{x} - \mathbf{x}_0),$$

and the trapping potential is given by

$$\mathrm{V}(\mathbf{x}) = \kappa(\mathbf{x} - \mathbf{x}_0)^2,$$

where κ denotes the (scalar) trap stiffness and $\mathbf{x}$-$\mathbf{x}_0$ the displacement from the particle's mean position $\mathbf{x}_0$. By explicitly considering the harmonic potential, the position distribution function $\mathrm{PDF}(\mathbf{x})$ becomes a Gaussian distribution with a standard deviation of

$$\sigma = \sqrt{\frac{k_b T}{2\kappa}}.$$

As the trap stiffness κ is directly proportional to the trap power P_trap[13], it follows for the full width at half maximum (FWHM) of the PDF that

$$FWHM = 2\sqrt{2\ln 2}\,\sigma \propto 1/\sqrt{P_\mathrm{trap}}.$$

For explicit consideration of the three-dimensional trap properties, the restoring force of the optical trap can be written in the form of

$$\mathbf{F}(\mathbf{x}) = -\boldsymbol{\kappa}\,(\mathbf{x} - \mathbf{x}_0),$$

where $\boldsymbol{\kappa}$ is the stiffness matrix in the coordinate system of the microscope. By an appropriate coordinate transformation, it is possible to approximate the stiffness matrix by a diagonal matrix, where the diagonal entries correspond to three (distinct) trap stiffness values in orthogonal spatial directions. By using a single, "perfect" trap focus the diagonal matrix $\boldsymbol{\kappa}$ consists of two equal elements indicating the lateral trap stiffness and one element indicating the axial trap stiffness. The axial stiffness is usually significantly lower than the lateral trap stiffness[14], causing position fluctuations of greater extent in the axial direction. If the effective trapping potential does not possess lateral rotational symmetry, e.g. caused by the shape of the

trapped object or the simultaneous use of multiple traps, the projection of the PDF to the lateral plane becomes an elliptical Gaussian distribution, i.e.

$$\mathrm{PDF}(x,y) = A\exp\left\{-\left(\frac{(x-x_0)\cdot\cos(\alpha) + (y-y_0)\cdot\sin(\alpha)}{\sqrt{2}\,\sigma_1}\right)^2 - \left(\frac{-(x-x_0)\cdot\sin(\alpha) + (y-y_0)\cdot\cos(\alpha)}{\sqrt{2}\,\sigma_2}\right)^2\right\},$$

where (x,y) is the object's position with the center position (x_0,y_0), α is the rotational angle of the ellipsoid axes relative to the coordinate system of the microscope, σ_1 and σ_2 are the independent standard deviations for orthogonal directions, and A is a normalization factor.

Supplementary Note 2: Validity of deconvolution approach

For the presented deconvolution approach to be valid, the measured *d*STORM localization distribution has to represent an image of the labelled structure convolved with the PDF. This will be given if either (i) each label is localized several times during the *d*STORM imaging process, or (ii) a high labeling density of the structure is achieved, thus, the distance between the labels is sufficiently small, i.e. on the order of the effective localization precision.

Though the first condition is difficult to measure for an individual experiment, Dempsey et al.[15] have found that each Alexa 647 fluorophore (the dye used in our experiments) could be localized during a mean number of 14 switching cycles under comparable buffer conditions (enzymatic oxygen removal using glucose oxidase and catalase plus using MEA as the reducing agent, though the MEA concentration was 10 mM instead of 100 mM). If single frame exposure times are shorter than the timespan of the fluorescence emission during a switching cycle, a fluorophore can be detected multiple times per switching cycle. This allows for an even higher number of localizations per fluorophore contributing to the reconstructed image. Additionally, one label, e.g. an antibody, might possibly be tagged by multiple fluorophores, further increasing the possible number of localizations per label. The number of localizations per label does, however, also depend on other experiment specific parameters, e.g. the number of frames recorded, and might therefore differ from the reported values. In any case, the condition of many localizations per label can in principle be met using Alexa 647.

From these considerations, we estimate the localization density to find the distance between the labels: The reconstruction of the bacterial cell shown in **Fig 3b** comprises 21,516 localizations detected in 15,285 raw images. The upper bound for the localization density is found by assuming exactly 1 localization for each fluorophore. The localizations cover an area of approx. 4 μm^2. Hence, the labeling density is at most $21516/4\ \mu m^{-2} = 5{,}379\ \mu m^{-2}$ which results in a lower bound for the mean distance between two fluorophores of about $\sqrt{(1/(5{,}379\ \mu m^{-2}))} = 13.6$ nm. Assuming 14 localizations for each fluorophore results in an overestimation as a second sequence of raw data could be recorded for this bacterium (Fig. 3d). Hence, the respective labeling density is at least $21{,}516/(4\times 14)\ \mu m^{-2} = 384\ \mu m^{-2}$, resulting in an upper bound for the mean distance between two fluorophores of about $\sqrt{(1/(384\ \mu m^{-2}))} = 51.0$ nm. The real mean distance between the detected labels will presumably lie somewhere in this interval between 13.6 nm and 51.0 nm. The comparison to the effective localization precision of 27.8 nm (**Supplementary Fig. 8a**) shows that it is on the order of the mean distance between the labels and, thus, the deconvolution approach is valid. Even if the real mean distance was close to the upper boundary of the interval and thus relatively high, this would indicate that individual

fluorophores had been localized multiple times. In this case, the first condition would be fulfilled and, again, the deconvolution approach is valid.

The labeling density of the bead edge can be estimated in a similar way. The reconstruction shown in **Fig. 1a** for a trap power of 5 mW consists of 15,236 localizations detected in 7,056 raw images recorded in approximately 220 s. As the diameter of the bead is 8.18 µm, its perimeter is 25.7 µm, and from the line profile shown in **Fig 1c** we estimate the localizations to be spread over a length of 0.5 µm orthogonal to the bead edge. Consequently, the localizations cover an area of 12.85 μm^2. As the bead presumably shows rotational Brownian motion during the imaging procedure (**Supplementary Fig 5**), the labels move and each position on the edge is detected just once rather than 14 times. These numbers yield an approximate labeling density of the bead edge of at most $15236/12.85\ \mu m^{-2} = 1186\ \mu m^{-2}$, giving a mean distance between two labels of about $\sqrt{(1/(1186\ \mu m^{-2}))} = 29$ nm. Again, this value is on the order of the effective localization precision of (41.3 ± 1.3) nm as determined by the approach of Endesfelder et al.[16] and confirms that the deconvolution approach is valid.

It is important to note that in our labeling strategy for the bacterial samples, the thymidine analogue EdU provides a relatively high concentration of binding sites for the fluorophores in the target nascent DNA that might be more difficult to achieve for other scenarios, e.g. antibody labeling approaches. Furthermore, organic dyes such as Alexa 647 are more favorable as they allow for multiple switching cycles in contrast to many types of photoactivatable proteins that might enable PALM imaging of optically trapped samples.

Supplementary Note 3: Experimental PDF generation

For PDF generation, a transmitted light image stack (**Fig. 1b**) is recorded prior or subsequent to the *d*STORM data acquisition. Compared to the microsphere experiment (**Fig. 1b**), where a single optical trap was used to hold each bead, the bacterium shows a strongly elliptical PDF (**Fig. 1b and Fig. 2d)**, yielding standard deviations for the 2D Gaussian of σ_1 = (43.6 ± 0.4) nm and σ_2 = (19.5 ± 0.2) nm. During displacements orthogonal to the long axis of the *E. coli* cell, a similarly high restoring force from both traps acts on the cell, while during displacements parallel to the long axis, the restoring force from one trap is significantly lower due to cell's rod-like shape (**Fig 1d**). This assumption is confirmed by the fact that the long axis of the PDF ellipse is co-located with the long axis of the bacterial cell. Moreover, these data show that the PDF not only depends on the optical trap properties, but also on the shape and alignment of the trapped object. That is why the PDF is not a property only of the setup, but has to be determined for each experiment separately. The beam used for fluorescence excitation and photoswitching also has an effect on the PDF. Usually, high intensities on the order of 0.5 to 5 mW/cm^2 are used[17], leading to non-negligible radiation pressure that may significantly displace the trapped object. As highly inclined and laminated optical sheet (HILO) excitation[18] is often used to obtain such high intensities, the specimen is displaced both in axial and lateral directions (**Supplementary Fig. 10**). Hence, the HILO beam is also switched on during the data acquisition for determination of the PDF, which leads to yet another issue: the bleaching of the fluorophores during this time. Accordingly, it is recommended to acquire the raw *d*STORM data first and record the transmitted light image stack in a second step. Regardless, it has emerged that the use of approximately 500 transmitted light image frames is sufficient to generate a PDF by 2D Gaussian fitting, which in this experiment resulted in a mean deviation of less than 1.4 % from the directly measured position distribution after acquiring 10,146 frames (**Supplementary Fig. 11**). By taking an effective acquisition time of 6 to 31 ms per frame into account, the measurements for the PDF can be conducted within 3 to 16 s, and could also be collected prior to the *d*STORM acquisition due to this relatively short time.

References for Supplementary Material

1. Thompson, R. E., Larson, D. R. & Webb, W. W. Precise nanometer localization analysis for individual fluorescent probes. *Biophys J* **82**, 2775-2783, doi:10.1016/S0006-3495(02)75618-X (2002).
2. Endesfelder, U., Malkusch, S., Fricke, F. & Heilemann, M. A simple method to estimate the average localization precision of a single-molecule localization microscopy experiment. *Histochem. Cell Biol.* **141**, 629-638 (2014).
3. Smith, C. S., Joseph, N., Rieger, B. & Lidke, K. A. Fast, single-molecule localization that achieves theoretically minimum uncertainty. *Nature Methods* **7**, 373-375, doi:10.1038/nmeth.1449 (2010).
4. Huang, B., Wang, W. Q., Bates, M. & Zhuang, X. W. Three-dimensional super-resolution imaging by stochastic optical reconstruction microscopy. *Science* **319**, 810-813, doi:10.1126/science.1153529|ISSN 0036-8075 (2008).
5. Egner, A. & Hell, S. W. in *Handbook of Biological Confocal Microscopy* (ed James B. Pawley) 404 (Springer, 2006).
6. Spahn, C., Endesfelder, U. & Heilemann, M. Super-resolution imaging of Escherichia coli nucleoids reveals highly structured and asymmetric segregation during fast growth. *J. Struct. Biol.* **185**, 243-249 (2014).
7. Wegel, E. *et al.* Imaging cellular structures in super-resolution with SIM, STED and Localisation Microscopy: A practical comparison. *Sci Rep-Uk* **6**, 2790, doi:10.1038/srep27290 (2016).
8. Schindelin, J. *et al.* Fiji – an Open Source platform for biological image analysis. *Nature Methods* **9**, 676-682 (2012).
9. Berg-Sørensen, K. & Flyvbjerg, H. Power spectrum analysis for optical tweezers. *Rev. Sci. Instrum.* **75**, 594-612 (2004).
10. Florin, E.-L., Pralle, A., Stelzer, E. H. K. & Hörber, J. K. H. Photonic force microscope calibration by thermal noise analysis. *Appl. Phys. A* **66**, 75-78 (1988).
11. Wördemann, M. *Structured Light Fields*. (Springer, 2012).
12. Dienerowitz, M., Mazilu, M. & Dholakia, K. Optical manipulation of nanoparticles: a review. *J. Nanophotonics* **2**, 021875 (2008).
13. Lee, W. M., Reece, P. J., Marchington, R. F., Metzger, N. K. & Dholakia, K. Construction and calibration of an optical Trap on a fluorescence optical microscope. *Nat Protoc* **2**, 3226-3238 (2007).
14. Bormouth, V. *et al.* Optical trapping of coated microspheres. *Opt Express* **16**, 13831-13844 (2008).
15. Dempsey, G. T., Vaughan, J. C., Chen, K. H., Bates, M. & Zhuang, X. W. Evaluation of fluorophores for optimal performance in localization-based super-resolution imaging. *Nature Methods* **8**, 1027-+, doi:10.1038/Nmeth.1768 (2011).
16. Endesfelder, U., Malkusch, S., Fricke, F. & Heilemann, M. A simple method to estimate the average localization precision of a single-molecule localization microscopy experiment. *Histochem Cell Biol* **141**, 629-638, doi:10.1007/s00418-014-1192-3 (2014).
17. van de Linde, S. *et al.* Direct stochastic optical reconstruction microscopy with standard fluorescent probes. *Nat Protoc* **6**, 991-1009, doi:10.1038/nprot.2011.336 (2011).
18. Tokunaga, M., Imamoto, N. & Sakata-Sogawa, K. Highly inclined thin illumination enables clear single-molecule imaging in cells. *Nature Methods* **5**, 159-161 (2008).
